# Zolbetuximab plus mFOLFOX6 for Claudin-18.2-Positive Advanced Gastric Cancer with Multiple Bone Metastases and Disseminated Intravascular Coagulation: A Case Report

**DOI:** 10.1159/cro/abzag021

**Published:** 2026-08-22

**Authors:** Taichi Tamura, Yusuke Kanemasa, Kumiko Fujita, Taro Sawabe, Ai Takahara, Shohei Nakamura, Toshihiro Okuya, Yu Yagi, Shota Kusuhara, Shinichiro Matsuda, Ryosuke Yoshioka, Shin-ichiro Horiguchi, Tatsu Shimoyama

**Affiliations:** a Department of Medical Oncology, Tokyo Metropolitan Cancer and Infectious Diseases Center Komagome Hospital, Tokyo, Japan; b Department of Neurosurgery, Tokyo Metropolitan Cancer and Infectious Diseases Center Komagome Hospital, Tokyo, Japan; c Department of Pathology, Tokyo Metropolitan Cancer and Infectious Diseases Center Komagome Hospital, Tokyo, Japan

**Keywords:** Advanced gastric cancer, Bone metastases, Claudin-18.2, Disseminated intravascular coagulation, mFOLFOX6, Zolbetuximab

## Abstract

**Introduction:**

Advanced gastric cancer (AGC) complicated by bone marrow carcinomatosis (BMC) and disseminated intravascular coagulation (DIC), for which there is currently no standard treatment, has a poor prognosis.

**Case Presentation:**

We report herein a 75-year-old male patient with claudin-18.2 (CLDN18.2)-positive, HER2-negative AGC who presented with multiple bone metastases and overt DIC, which raised the index of suspicion for BMC. Although his poor performance status (PS) was attributed primarily to cancer-related pain and coagulopathy, systemic chemotherapy was started with mFOLFOX6. Early in the treatment course, acute-on-chronic subdural hematoma developed in the presence of severe thrombocytopenia, and the patient underwent burr-hole drainage after receiving intensive transfusion support. Once CLDN18.2 positivity was confirmed, zolbetuximab was added from cycle 2. The DIC parameters improved and stabilized. Hematological recovery followed, the primary lesion showed regression on imaging, and his PS improved modestly. He received seven cycles of zolbetuximab plus mFOLFOX6. However, his PS did not recover sufficiently to permit the continuation of outpatient infusional chemotherapy, prompting a treatment transition to S-1 monotherapy followed by best supportive care. The patient survived 233 days after the initial diagnosis.

**Conclusion:**

The present case demonstrated that zolbetuximab-containing therapy combined with multidisciplinary management can be a feasible option for selected patients with CLDN18.2-positive AGC complicated by DIC and clinically suspected BMC.

## Introduction

Bone marrow carcinomatosis (BMC), defined as diffuse, metastatic infiltration of the marrow cavity, is rare. The prevalence of biopsy-confirmed bone marrow metastasis in advanced gastric cancer (AGC) is 2.4% [1]. In a previous report, 3.73% of 964 AGC patients presented with concurrent disseminated intravascular coagulation (DIC) and diffuse bone marrow metastasis [2]. The prognosis of these patients was dismal; DIC occurs as a complication in >80% of BMC cases, and pooled data indicate median survival <1 month without chemotherapy [3, 4]. Although systemic therapy can extend survival to 2–7 months [3, 5], no standard regimen has yet been established.

The therapeutic landscape of AGC shifted in 2023 with the advent of zolbetuximab, a monoclonal antibody targeting claudin-18.2 (CLDN18.2). In two phase III trials, SPOTLIGHT (zolbetuximab + mFOLFOX6) and GLOW (zolbetuximab + CAPOX), zolbetuximab-based therapy significantly prolonged progression-free survival and overall survival versus chemotherapy alone, e.g., in SPOTLIGHT, the hazard ratio for disease progression or death was 0.75 (95% confidence interval: 0.60–0.94) [6, 7]. However, the efficacy and feasibility of zolbetuximab against AGC complicated by overt DIC and clinically suspected BMC are unclear.

Herein, we report a case of CLDN18.2-positive AGC with multiple bone metastases and overt DIC, for which zolbetuximab was added to mFOLFOX6 after the first chemotherapy cycle. Early in the treatment course, an intracranial hemorrhage requiring emergent neurosurgical intervention occurred, temporarily interrupting the systemic therapy. However, subsequent, coordinated management by a team consisting of medical oncologists, hematologists, and neurosurgeons enabled the chemotherapy to be resumed. As a result, the patient experienced sustained, hematological recovery, as indicated by improvement in the platelet, fibrinogen, and D-dimer values. This case illustrates both the potential feasibility of CLDN18.2-targeted therapy for this critically ill population and the practical constraints of such an approach, including the risk of unpredictable bleeding, need for intensive multidisciplinary support, and the uncertainty of diagnosing BMC without histological confirmation.

## Case Presentation

A 75-year-old male patient with no significant medical history initially presented to a previous hospital with lower back pain. His evaluation included spinal magnetic resonance imaging, which revealed multiple, round, low-intensity areas in the thoracic and lumbar vertebrae on T1-weighted imaging, strongly suggesting multiple bone metastases (Fig. 1a). Computed tomography revealed slight wall thickening with contrast enhancement in the gastric antrum and pyloric region, which were consistent with the primary lesion (Fig. 1b). The scan demonstrated no clear evidence of a visceral metastasis or significant lymphadenopathy and only a small amount of ascites in the pelvis (Fig. 1c). Furthermore, upper gastrointestinal endoscopy identified an irregular, ulcerative lesion on the lesser curvature of the gastric antrum (Fig. 1d), a biopsy of which confirmed poorly differentiated adenocarcinoma (Fig. 2a). The patient was subsequently referred to our hospital for further management. He had not received prior systemic anticancer therapy, radiotherapy, or surgery for gastric cancer before referral.

**Fig. 1.**
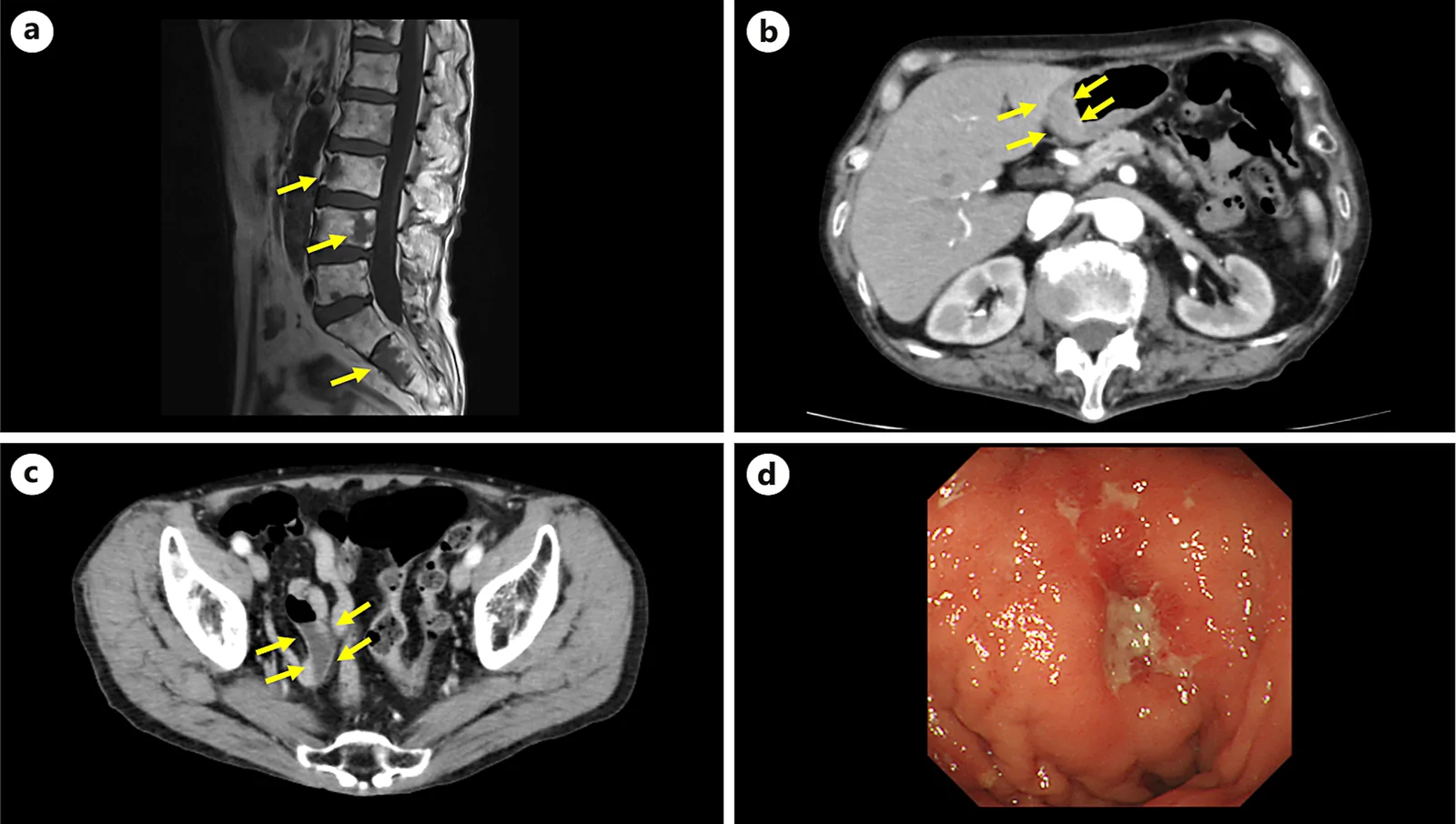
**a** Sagittal T1-weighted magnetic resonance imaging of the thoracolumbar spine revealed multiple, round to patchy, low-intensity lesions involving vertebral bodies, pedicles, and spinous processes (representative lesions indicated by yellow arrows), which were highly suggestive of extensive bone marrow involvement. No epidural extension or spinal cord compression was observed. **b** Axial contrast-enhanced computed tomography (CT) imaging demonstrated slight wall thickening with enhancement of the gastric antrum and pyloric region (yellow arrows), which were consistent with the primary lesion. No definite, visceral metastases or significant lymphadenopathy was identified. **c** Axial contrast-enhanced CT imaging at the pelvic level demonstrated a small amount of ascites (yellow arrows). **d** Upper gastrointestinal endoscopy demonstrated an irregular, ulcerative lesion on the lesser curvature of the gastric antrum.

**Fig. 2.**
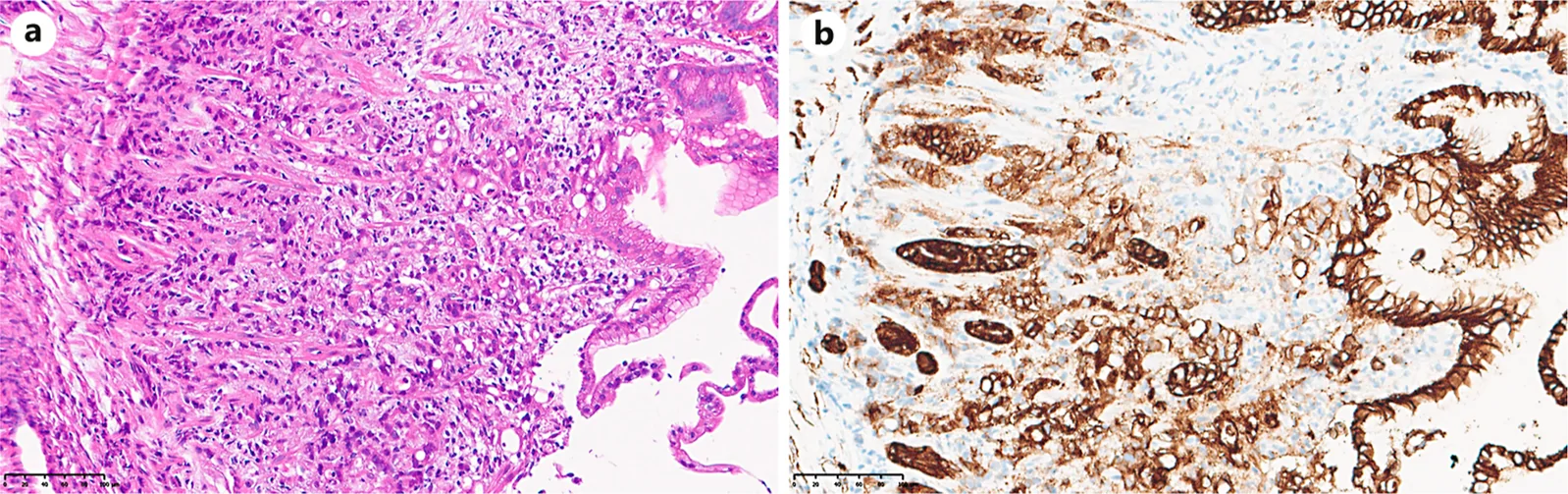
**a** Hematoxylin and eosin staining demonstrated poorly differentiated adenocarcinoma. **b** Immunohistochemistry for CLDN18.2 revealed membranous staining in approximately 80% of the tumor cells.

Upon admission to our department, his Eastern Cooperative Oncology Group (ECOG) performance status (PS) was 4. On admission, laboratory findings indicated marked coagulopathy, as indicated by platelet count 58,000/μL, prothrombin time 17.0 s, fibrinogen 94 mg/dL, and fibrinogen degradation products 189.3 μg/mL. These findings fulfilled the diagnostic criteria for DIC (International Society on Thrombosis and Haemostasis [ISTH] score: 5), and AGC accompanied by multiple bone metastases and DIC, features highly suggestive of BMC, was diagnosed.

While a PS of 4 is typically a contraindication for systemic chemotherapy, the patient’s severe functional impairment was thought to be primarily due to uncontrolled pain from the bone metastases and potentially reversible. The goal of chemotherapy was thus palliative, aiming to alleviate pain and improve his functional status. A formal patient-reported perspective was not collected. However, the patient expressed a strong desire to continue active anticancer therapy despite persistent functional impairment. After a thorough discussion with the patient and his family about the significant risks versus the potential for symptomatic improvement, a shared decision was made to proceed with systemic chemotherapy in conjunction with aggressive pain management.

Pending biomarker test results, first-line chemotherapy with the mFOLFOX6 regimen was begun on the day after admission (cycle 1, day 1). Given the patient’s critical condition, severe thrombocytopenia, and ECOG PS of 4, the first cycle of mFOLFOX6 was dose-modified. Oxaliplatin was reduced from 85 mg/m^2^ to 65 mg/m^2^, the bolus fluorouracil was omitted, and continuous fluorouracil was reduced from 2,400 mg/m^2^ to 2,000 mg/m^2^. However, during the same night, the patient experienced delirium and removed the intravenous line, prematurely interrupting the continuous fluorouracil infusion; the delivered dose was estimated to be approximately 10% of the planned dose based on the infusion duration. On day 4 of the cycle, his consciousness status acutely deteriorated to a Glasgow Coma Scale (GCS) score of 10 (E3V4M3). Urgent head computed tomography revealed a right-sided acute-on-chronic subdural hematoma with a midline shift (online suppl. Fig. S1; for all online suppl. material, see https://doi.org/10.1159/cro/abzag021). However, immediate neurosurgical intervention was precluded by severe thrombocytopenia. Burr-hole drainage was planned with the proviso that the platelet count be maintained above 50,000/μL via transfusions. Following frequent platelet transfusions and the potential effect of the initial, partial dose of mFOLFOX6, the platelet count marginally rose just above the threshold, allowing burr-hole drainage to be performed on day 7. His consciousness status improved promptly after the surgery.

Subsequent biomarker analyses revealed HER2 immunohistochemistry score 1+ (interpreted as HER2-negative), PD-L1 combined positive score (CPS; clone 22C3) 1–10, and CLDN18.2 expression in 80% of the tumor cells (Fig. 2b). Immunohistochemically assessed MMR status demonstrated proficient mismatch repair. Microsatellite instability testing using the FALCO kit confirmed microsatellite stability. Accordingly, the regimen was switched to zolbetuximab plus mFOLFOX6 beginning with cycle 2 (C2). Zolbetuximab was administered at 1,200 mg, corresponding to approximately 800 mg/m^2^. The drug was diluted to 2 mg/mL and infused with stepwise escalation of the infusion rate: 75 mL/h for 30 min, 150 mL/h for 30 min, and then 300 mL/h. Prophylactic antiemetic therapy included aprepitant, olanzapine, palonosetron, dexamethasone, and chlorpheniramine. The first infusion was completed without nausea, vomiting, anorexia, infusion interruption, or infusion rate reduction. To manage the severe pain from his spinal metastases, the patient also received palliative radiotherapy (8 Gy in a single fraction), and zoledronic acid was concurrently administered as a bone-modifying agent. The combination of these treatments resulted in remarkable clinical improvement. His pain was significantly alleviated, his ECOG PS improved to 3, and he became ambulatory with a walker. Concurrently, his DIC-related laboratory parameters, including the platelet count, showed a sustained improvement, and he was able to discontinue both platelet and red blood cell transfusions (Fig. 3).

**Fig. 3.**
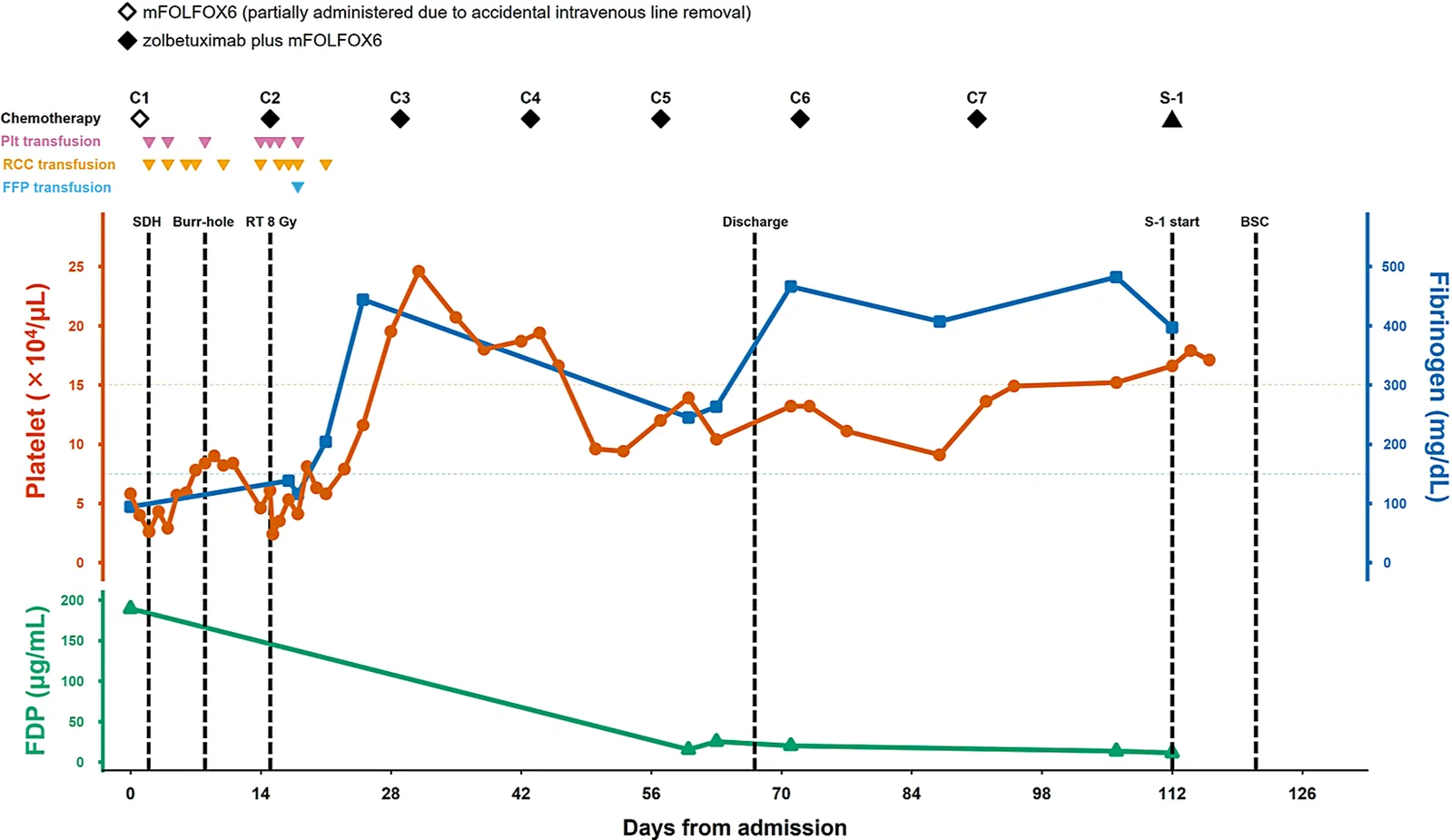
Time course of the platelet count (orange line, left y-axis), fibrinogen (blue line, right y-axis), and FDP (green line, lower panel) from admission (day 0). Chemotherapy cycles are indicated in the upper track: the open diamond (C1) represents mFOLFOX6 alone, which was partially administered due to an accidental removal of the intravenous line. The closed diamonds (C2 through C7) represent zolbetuximab plus mFOLFOX6, which was initiated after confirming CLDN18.2 positivity. The triangle represents S-1 monotherapy. Transfusion records are indicated by upside-down triangles (pink: platelet concentrate; orange: red blood cell concentrate; light blue: fresh frozen plasma). The following clinical events are indicated by the vertical dotted lines: SDH, subdural hematoma onset (day 4); burr-hole, burr-hole drainage (day 7); RT 8 Gy, palliative radiotherapy to the thoracolumbar spine for bone metastasis pain (8 Gy in a single fraction); discharge, first hospital discharge (day 66); S-1 start, initiation of S-1 monotherapy; BSC, transition to best supportive care. The horizontal dashed lines indicate reference levels (platelet 15 × 10^4^/μL; fibrinogen 150 mg/dL). Cycles 1 through 5 were administered during a single, continuous hospitalization; subsequent cycles were administered during brief re-admissions, reflecting the patient’s persistent, functional limitations. BSC, best supportive care; C, cycle; DIC, disseminated intravascular coagulation; FDP, fibrin/fibrinogen degradation products; mFOLFOX6, modified folinic acid, 5-fluorouracil, and oxaliplatin; RT, radiotherapy; SDH, subdural hematoma.

The patient completed the first five cycles of zolbetuximab plus mFOLFOX6 during a single, continuous hospitalization and was first discharged on day 66. Radiological assessment by computed tomography after five cycles of systemic therapy showed improvement of the gastric wall thickening suspected to correspond to the primary lesion. Although his PS had improved from 4 to 3 after the initial treatment, no further functional recovery was observed thereafter. The subsequent two cycles (C6 and C7) were administered during brief re-admissions; sustained outpatient management was not possible given his persistent PS impairment. Because the patient expressed a strong desire to receive active therapy, the choice was made to de-escalate to oral S-1 monotherapy. However, he tolerated the treatment poorly primarily because of mucosal toxicity and fatigue, and S-1 monotherapy was discontinued after one cycle. After shared decision-making with the patient and his family, the treatment goal was transitioned to best supportive care. The patient died 233 days after the initial diagnosis. This case report was prepared in accordance with the CARE guidelines, and the completed CARE checklist is provided as online supplementary material.

## Discussion

This case illustrates that even when AGC is complicated by clinically suspected BMC and overt DIC – historically, a near-fatal combination – an integrated approach can achieve meaningful disease stabilization and improve the patient’s overall condition. The urgent burr-hole drainage of the hematoma resolved the life-threatening intracranial pressure, stabilizing the patient for the zolbetuximab-based therapy. The administration of zolbetuximab plus mFOLFOX6, which was supported by intensive transfusion management, resulted in a sustained recovery of the platelet count and normalization of the DIC parameters. The sequence of the patient’s treatments underscores the importance of close, multidisciplinary coordination when malignancy-related coagulopathy precipitates life-threatening bleeding. Nevertheless, the clinical course also illustrates a practical limitation of this approach. Despite the biological response, the patient’s failed to recover sufficiently for outpatient infusional chemotherapy to be administered, and the regimen was sustainable only via repeated hospitalizations. In critically ill patients with BMC and DIC, intensive, multi-agent, intravenous therapy may achieve disease control yet fall short of achieving long-term recovery of ambulation, an important consideration when weighing treatment intensity against quality of life.

BMC is a devastating complication of gastric cancer and is associated with a dismal prognosis, a high burden of skeletal-related events, and severe hematological disorders, including DIC [8]. The associated coagulopathy frequently leads to fatal bleeding, with one study reporting intracranial hemorrhage as the cause of death in 25% of patients with BMC [3]. The underlying pathophysiology involves a vicious cycle: in BMC, tumor-derived cytokines (e.g., PTHrP, IL-6) trigger RANK-L-driven osteoclast activation, liberating matrix-sequestered growth factors (TGF-β, IGFs) that fuel further tumor growth and perpetuate coagulopathy [8]. Disrupting this vicious cycle is therefore a key therapeutic goal. The current method of management is multimodal. Systemic chemotherapy can extend survival to a greater extent than best supportive care in gastric cancer with BMC, particularly when DIC is controlled, although the median overall survival is still only a few months [3, 5]. Bone-modifying agents, such as zoledronic acid, are a key component of management aimed primarily at reducing skeletal-related events, although they have also demonstrated a survival benefit in some retrospective cohorts [9–11]. Evidence specific to BMC is limited to small case series and case reports. Furthermore, these approaches often provide limited benefits, thus highlighting the need for novel therapies that can more effectively break the pathological cycle.

Zolbetuximab binds with the tight junction protein CLDN18.2, thereby mediating complement-dependent, antibody-dependent cellular cytotoxicity against CLDN18.2-positive tumor cells [12, 13]. Although there are few data on zolbetuximab’s ability to penetrate to bone marrow, targeted therapies have been associated with hematological improvement in other malignancies complicated by BMC and DIC, for example, crizotinib in non-small cell lung cancer [14], palbociclib plus letrozole in breast cancer [15], and sintilimab plus chemotherapy and trastuzumab in gastric cancer [16]. In our case, however, the respective contribution of cytotoxic chemotherapy, zolbetuximab, and transfusion support to the patient’s hematological recovery cannot be separated, and prospective data on patients with BMC-associated coagulopathy are needed to clarify the role of CLDN18.2-targeted therapy in this context.

Anti-PD-1-based chemotherapy could also have been considered as a rational first-line option. However, the present tumor was HER2-negative and proficient mismatch repair/microsatellite stability, with diffuse-type adenocarcinoma and signet-ring cell features, and the PD-L1 CPS was 1–10, which did not clearly identify a high PD-L1 subgroup. Previous studies suggest that the benefit of immune checkpoint inhibition may be less consistent in diffuse-type gastric cancer [17], and a retrospective analysis of first-line PD-1 inhibitor plus chemotherapy showed lower response rates and shorter progression-free survival in diffuse-type tumors, particularly in the PD-L1 CPS <5 population [18]. Conversely, the tumor showed CLDN18.2 positivity in 80% of tumor cells, meeting the threshold used in pivotal zolbetuximab trials. Therefore, zolbetuximab was selected as a CLDN18.2-directed add-on therapy to mFOLFOX6 after CLDN18.2 positivity was confirmed. This choice does not imply that zolbetuximab induces faster responses than anti-PD-1-based chemotherapy, and the optimal selection, sequencing, or combination of these agents remains to be established.

Gastric cancer with BMC and DIC, which is considered to have a particularly poor prognosis, is more common in the diffuse type [8, 19]. In fact, the diffuse type reportedly occurs in 30.0% of cases of gastric cancer with bone metastasis [19] and 86% of those with DIC [20]. Furthermore, multiple studies have reported significantly higher CLDN18.2 positivity in diffuse-type than in intestinal-type tumors, for example, 48.3% and 38.8% of the respective type in the pivotal SPOTLIGHT/GLOW trials [21], which do not differ significantly from the figures of 42.5% vs. 28.9% and 38.5% vs. 24.9% for the respective type reported in other cohorts [22, 23]. This consistent enrichment of CLDN18.2 in diffuse-type tumors provides a biological rationale for CLDN18.2-targeted therapy in this high-risk population and has been reported across cohorts [21, 24]. However, no established evidence directly links CLDN18.2 expression to DIC or disseminated bone marrow involvement. Rather than a direct mechanistic link, the apparent co-occurrence of CLDN18.2 positivity and DIC with suspected marrow involvement in this setting may instead reflect their shared association with diffuse-type histology because gastric cancer-associated disseminated carcinomatosis of the bone marrow with DIC has classically been reported in poorly differentiated adenocarcinoma, signet-ring cell carcinoma, and mucin-producing adenocarcinoma [4, 5]. Therefore, any relationship between CLDN18.2 positivity and DIC with suspected marrow involvement should be regarded as indirect and hypothesis-generating. This single case cannot support a causal association.

This report has several limitations. First, as a single case report, its findings regarding the efficacy and safety of this treatment regimen cannot be generalized. Second, BMC was diagnosed clinically on the basis of MRI and laboratory data findings rather than on the pathological findings of a bone biopsy. Bone scintigraphy was not performed. Skeletal involvement was evaluated mainly by spinal MRI, which showed multiple vertebral lesions, together with laboratory findings suggestive of extensive bone involvement. However, the patient’s subsequent response to systemic chemotherapy, including the improvement of his clinical symptoms and the sustained normalization of the DIC parameters, was consistent with this clinical diagnosis.

In conclusion, zolbetuximab plus chemotherapy, when combined with intensive multidisciplinary support, may be a feasible initial treatment option for CLDN18.2-positive AGC complicated by clinically suspected BMC and DIC. In the present patient, this multidisciplinary, biomarker-directed approach was associated with disease control and a survival of 233 days from the initial diagnosis, longer than the median survival historically reported for this condition. However, achieving sustained recovery of ambulation remains a practical challenge in patients with this disease. Data from more cases are needed to clarify the efficacy and long-term applicability of this approach.

## Acknowledgment

We would like to thank James R. Valera for his assistance with editing this manuscript.

## Statement of Ethics

Written informed consent was obtained from the patient for publication of the details of their medical case and any accompanying images. Ethical approval is not required for this study in accordance with local or national guidelines.

## Conflict of Interest Statement

The authors declare no conflicts of interest.

## Funding Sources

This study was not supported by any sponsor or funder.

## Author Contributions

Taichi Tamura and Yusuke Kanemasa drafted the manuscript. Taichi Tamura, Yusuke Kanemasa, Kumiko Fujita, Taro Sawabe, Ai Takahara, Shohei Nakamura, Toshihiro Okuya, Yu Yagi, Shota Kusuhara, Shinichiro Matsuda, Ryosuke Yoshioka, Shin-ichiro Horiguchi, and Tatsu Shimoyama participated in the patient’s treatment. All the authors have read and approved the final manuscript.

## Data Availability

All data generated or analyzed during this study are included in this article and its online supplementary material. No additional data are available.
